# Prophylactic Catechin-Rich Green Tea Extract Treatment Ameliorates Pathogenic Enterotoxic *Escherichia coli*-Induced Colitis

**DOI:** 10.3390/pathogens10121573

**Published:** 2021-12-02

**Authors:** Jeong-Won Kim, Chang-Yeop Kim, Jin-Hwa Kim, Ji-Soo Jeong, Je-Oh Lim, Je-Won Ko, Tae-Won Kim

**Affiliations:** 1 College of Veterinary Medicine (BK21 FOUR Program), Chungnam National University, 99 Daehak-ro, Daejeon 34131, Korea; lilflflb@gmail.com (J.-W.K.); 963ckdduq@gmail.com (C.-Y.K.); jinhwa92696@gmail.com (J.-H.K.); jisooj9543@gmail.com (J.-S.J.); 2 College of Veterinary Medicine (BK21 Plus Project Team), Chonnam National University, 77 Yongbong-ro, Buk-gu, Gwangju 61186, Korea; dvmljo@naver.com

**Keywords:** pathogenic *Escherichia coli*, *Camellia sinensis*, catechin, annexin A1, colitis

## Abstract

In this study, we explored the potential beneficial effects of green tea extract (GTE) in a pathogenic *Escherichia coli* (F18:LT:STa:Stx2e)-induced colitis model. The GTE was standardized with catechin and epigallocatechin-3-gallate content using chromatography analysis. Ten consecutive days of GTE (500 and 1000 mg/kg) oral administration was followed by 3 days of a pathogenic *E. coli* challenge (1 × 10^9^ CFU/mL). In vitro antibacterial analysis showed that GTE successfully inhibited the growth of pathogenic *E. coli*, demonstrating over a 3-fold reduction under time- and concentration-dependent conditions. The in vivo antibacterial effect of GTE was confirmed, with an inhibition rate of approximately 90% when compared to that of the *E. coli* alone group. GTE treatment improved pathogenic *E. coli*-induced intestinal injury with well-preserved epithelial linings and villi. In addition, the increased expression of annexin A1 in GTE-treated jejunum tissue was detected, which was accompanied by suppressed inflammation-related signal expression, including TNFA, COX-2, and iNOS. Moreover, proliferation-related signals such as PCNA, CD44, and Ki-67 were enhanced in the GTE group compared to those in the *E. coli* alone group. Taken together, these results indicate that GTE has an antibacterial activity against pathogenic *E. coli* and ameliorates pathogenic *E. coli*-induced intestinal damage by modulating inflammation and epithelial cell proliferation.

## 1. Introduction

*Escherichia coli (E. coli)* is normally found in intestinal flora and typically colonizes the gastrointestinal tract. As a nonpathogenic microbiota, *E. coli* participates in the biotransformation of xenobiotics and the synthesis of organic molecules [1]. However, *E. coli* presents special pathogenic factors that were reported to cause a wide spectrum of infectious diseases [2]. *E. coli* is a common cause of diarrheal disease worldwide and it is estimated that 200 million people are affected by it every day. Moreover, increased antibiotic resistance in *E. coli* contributes to morbidity, mortality, and has significant impacts on the health and social implications associated with infection [3].

Previous studies categorized pathogenic *E. coli* by its pathotype, and, among the groups, enteropathogenic *E. coli*, enterohemorrhagic *E. coli*, and enterotoxigenic *E. coli* (ETEC) were reported to cause disease in both humans and animals using many of the common virulence factors [4]. Pathogenic *E. coli* adhere to specific host cells with adhesins, also called fimbriae, to colonize and secrete various toxins such as the Shiga toxin (Stx), as well as heat-labile (LT) and heat-stable (ST) toxins to compromise cell function [5]. Pathogenic *E. coli*-induced colonic damage is accompanied with intense inflammation [6]. In addition to endotoxins, lipopolysaccharides (LPS) and flagella in *E. coli* trigger a potent inflammatory cytokine cascade and stimulate annexin A1 (Anex1) expression, which is an anti-inflammatory protein that inhibits inflammatory cell transmigration and phospholipase A2 activation [4,7].

Currently, it is not uncommon to report bacteria with existing antibiotic resistance, which have become a global threat, and pathogenic *E. coli* are also known to exhibit such resistance [8]. In this regard, the discovery of novel therapeutic approaches and the use of natural antimicrobial compounds is regarded as a potential alternative to reduce the overuse of antibiotics and the emergence of antibiotic resistance [9].

The tea plant, *Camellia sinensis*, is one of the major sources gaining considerable attention because of its safety and therapeutic potential. In the last decade, numerous studies recognized polyphenol components from *C. sinensis* as active ingredients and explored possible defensive activities against diverse pathogens, including fungi, viruses, and bacteria [8,10,11]. Polyphenols consist of two general classes: flavonoids and phenolic acid, and catechin. The latter, which belongs to the flavonoid class, is a major constituent of *C. sinensis*. Among the catechins, (-)-epigallocatechin-3-gallate (EGCG) is the most abundant catechin (approximately 60%) found in *C. sinensis* [11]. Previous studies revealed the antimicrobial activity of various catechin compounds against various pathogens, and the antimicrobial activity of epicatechin and EGCG was reported more than other catechin compounds (Table 1). In addition, apart from the antibacterial activity of catechins, catechin compounds were reported to exert anti-inflammatory effects by inhibiting the Toll-like receptor 4/nuclear factor κB signaling pathway and inflammatory cytokines [12,13]. In this study, the preventive effect of standardized catechin-rich green tea extract (GTE) against wild-type pathogenic *E. coli*-induced colitis was explored by evaluating the antibacterial and anti-inflammatory activity of GTE.

## 2. Results

### 2.1. Catechin Content in the GTE

**Figure 1:**
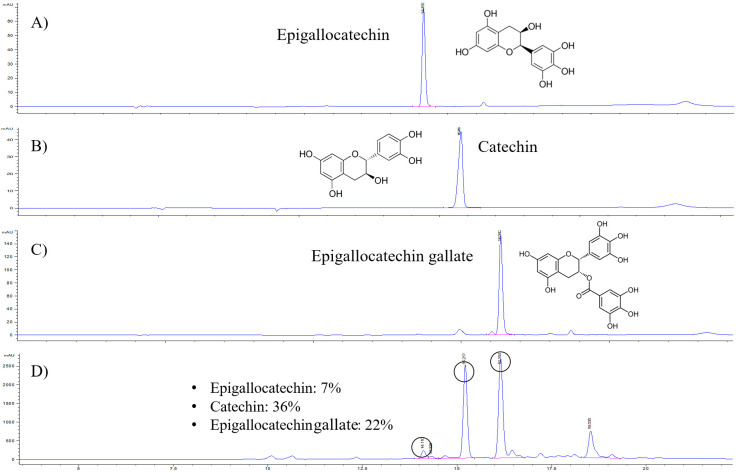
HPLC fingerprinting of green tea extract (GTE) and chromatogram of reference standards. HPLC-UV analysis was performed to determine (**A**) epigallocatechin, (**B**) catechin, (**C**) epigallocatechin gallate, and (**D**) GTE compounds.

In the present study, a high-performance liquid chromatography–ultraviolet (HPLC–UV) analytical method was established and applied to determine the levels of epigallocatechin, catechin, and EGCG in the GTE for quality control (Figure 1). Serial concentrations of all standard compounds were analyzed to obtain the standard curves. The linear range for all compounds was set at 1–100 μg/mL, with r^2^ = 0.999. The limits of detection and quantification for all compounds were 0.3 and 1 μg/mL, respectively. The three analytes were fully separated within 20 min. A representative HPLC chromatogram is shown in Figure 1. The retention times for epigallocatechin, catechin, and EGCG were approximately 14.1, 15.5, and 16.9 min, respectively. The GTE contained 7% epigallocatechin, 36% catechin, and 22% EGCG.

### 2.2. Antibacterial Effect of GTE against Pathogenic E. coli

**Figure 2:**
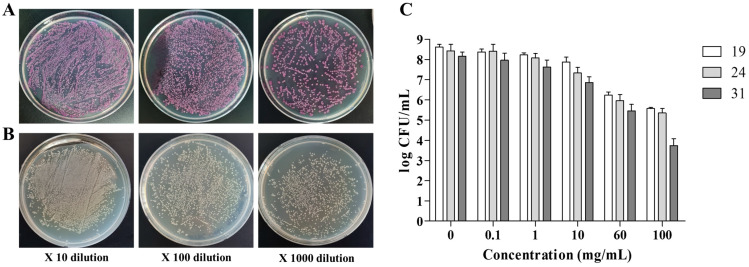
Streaked pathogenic *Escherichia coli* on selective agar, and antibacterial analysis. (**A**) ChormoSelect agar plate. (**B**) Nutrient agar plate. (**C**) Bacterial count.

The antibacterial effect of GTE against pathogenic *E. coli* was assessed using the microdilution and agar plate spreading method. The growth of pathogenic *E. coli* on ChromoSelect selective agar was confirmed before analysis. The minimum inhibitory concentration of GTE against pathogenic *E coli.* was 40 mg/mL, which prevented the visible growth of bacteria. In addition, the reduction in pathogenic *E coli.* after GTE treatment was evaluated and the results were expressed as log colony forming units (CFU)/mL (Figure 2). After GTE treatment for different times, the bacterial count of pathogenic *E. coli* decreased in a time- and concentration-dependent manner. Moreover, at a high concentration (>60 mg/mL), an over 3-log reduction was observed in the CFU value when compared to that of the control group.

### 2.3. Effect of GTE on Pathogenic E. coli-Induced Intestinal Injury

**Figure 3:**
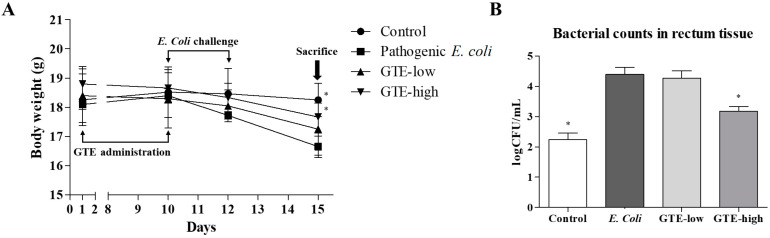
Experimental procedure and body weight changes after pathogenic *Escherichia coli* challenge in green tea extract (GTE)-treated or untreated mice (**A**), and bacterial counts in rectum tissue (**B**). * Significantly different from pathogenic *E. coli*, *p* < 0.05.

The mice were challenged with pathogenic *E. coli* after 10 days of GTE administration. After three oral bacterial challenges, the body weight of challenged mice gradually decreased and that of the *E. coli* alone group showed the lowest body weight, which was approximately 9% less than that of the normal control group (Figure 3). Although the GTE-treated groups were also found to have decreased body weight after the bacterial challenge, the decrease was relatively small when compared to the *E. coli* alone group.

Moreover, GTE administration suppressed the bacterial count in the intestine. The bacterial count was performed in the rectum tissue, and the total *E. coli* count was examined using selective agar. Ten consecutive days of GTE treatment suppressed bacterial counts in the intestine in a dose-dependent manner. The CFU values were reduced by 26% and 94% in the low- and high-dose groups, respectively, when compared to those in the *E. coli* alone group.

### 2.4. Effect of GTE on Pathogenic E. coli-Induced Alterations in the Jejunum

**Figure 4:**
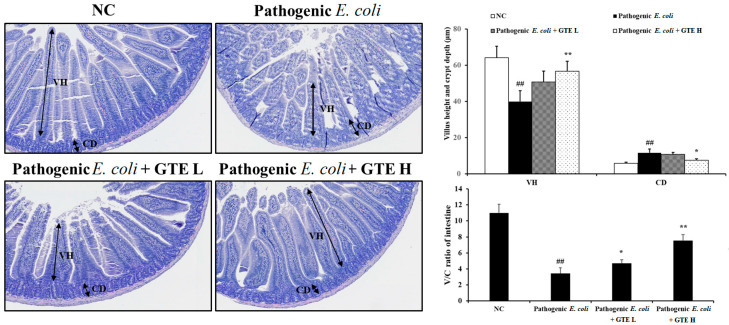
Effects of green tea extract (GTE) on enterotoxigenic *Escherichia coli* (ETEC)-induced histopathological alterations in the intestine. Values are presented as means ± SD (*n* = 6). ## Significantly different from normal control (NC), *p* < 0.01; * and ** significantly different from pathogenic *E. coli*, *p* < 0.05 and <0.01, respectively. VH, villus height; CD, crypt depth; V/C ration, callus height/crypt depth.

The beneficial antibacterial effect of GTE was also supported by the histopathological findings (Figure 4). The normal control group mice showed well-preserved villi with intact mucosal lining and a crypt layer in the jejunum tissue. In contrast, the *E. coli*-challenged groups showed a relatively shortened villi length with slight hyperplastic changes in the crypt, and this pathological change was improved by GTE treatment. The villi height/crypt depth ratio significantly increased after high-dose GTE treatment.

### 2.5. Effect of GTE on Annexin A1 Expression in Pathogenic E. coli-Challenged Mice Intestine

**Figure 5:**
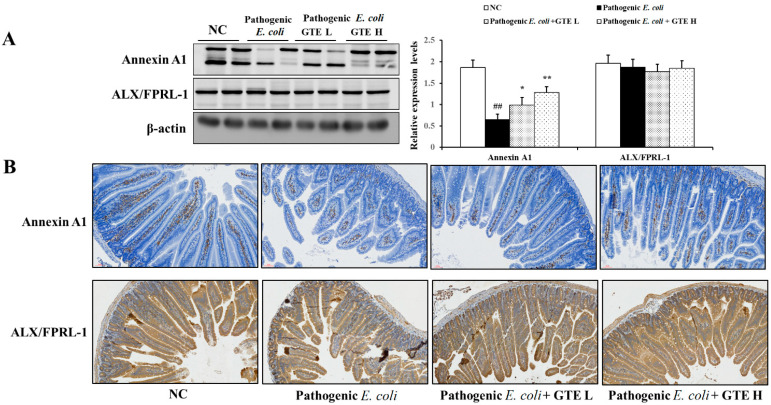
Effects of green tea extract (GTE) on expression of Annexin A1 and FPRL1/RFP. (**A**) Western blot analysis of Annexin A1 and FPRL1/RFP (loading control: β-actin). (**B**) Representative photographs of immunohistochemical analysis of Annexin A1 and FPRL1/RFP. Values are presented as means ± SD (*n* = 6). ## Significantly different from normal control (NC), *p* < 0.01; * and ** significantly different from pathogenic *Escherichia coli*, *p* < 0.05 and <0.01, respectively.

The protein levels of Anex1 and ALX/FPRL-1 were confirmed by Western blot analysis (Figure 5A). The *E. coli* challenge group showed low basal levels of Anex1 expression. However, GTE treatment enhanced Anex1 expression by approximately 1.5–2 times that of the *E. coli* alone group after 500 and 1000 mg/kg GTE treatment, respectively. GTE-induced Anex1 expression was also confirmed by immunohistochemical (IHC) evaluation (Figure 5B). The GTE-treated group showed restored and localized Anex1 expression in the lamina propria. The expression of ALX/FPRL-1 was relatively stable and unchanged in all experimental groups.

### 2.6. Effect of GTE on Inflammation and Proliferation-Related Signal Expression

**Figure 6:**
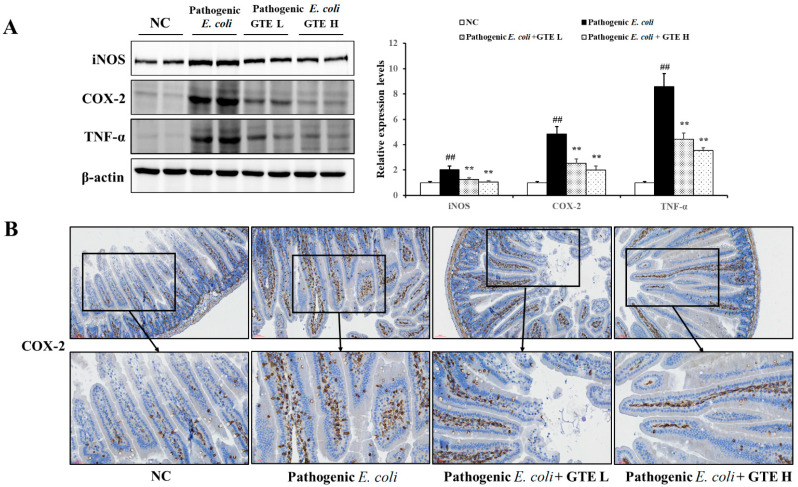
Effects of green tea extract (GTE) on the expression of pathogenic *Escherichia coli*-induced inflammatory proteins. (**A**) Western blot analysis of iNOS, COX-2, and TNF-α (loading control: β-actin). (**B**) Representative photographs of immunohistochemical analysis of COX-2. Values are presented as means ± SD (*n* = 6). ## Significantly different from normal control (NC), *p* < 0.01; ** significantly different from pathogenic *E. coli*, *p* < 0.01.

The effect of GTE on *E. coli*-induced inflammatory signals was explored by assessing the tumor necrosis factor-α (TNF-α), cyclooxygenase-2 (COX-2), and inducible nitric oxide synthase (iNOS) expression (Figure 6A). As expected, the *E. coli* challenge evidently enhanced the inflammation-related signals, and TNF-α was the most affected among the tested signal proteins. *E. coli*-induced, inflammation-related signal proteins were significantly suppressed in the GTE-treated group. Reduced inflammation-related signals were also detected in the COX-2 IHC evaluation (Figure 6B). *E. coli* challenge markedly increased COX-2 expression in the villus lining and lamina propria of the jejunum. However, this upregulated COX-2 expression was resolved in the GTE-treated group when compared to that in the *E. coli* alone group.

**Figure 7:**
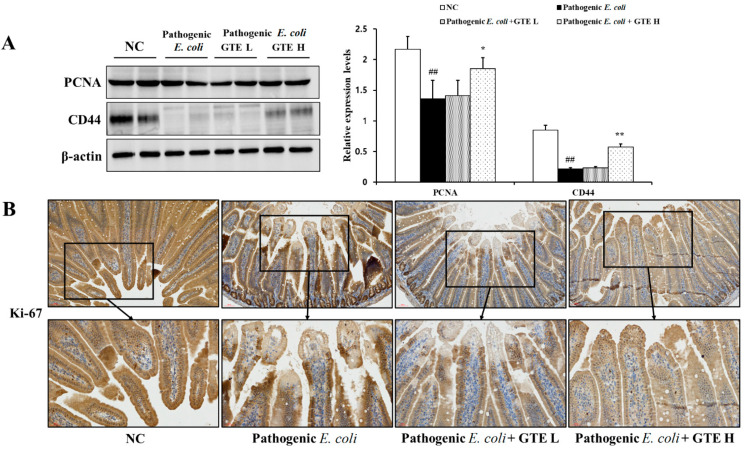
Effects of green tea extract (GTE) on expression of proliferative proteins. (**A**) Western blot analysis of PCNA and CD44 (loading control: β-actin). (**B**) Representative photographs of immunohistochemical analysis of Ki-67. Values are presented as means ± SD (*n* = 6). ## Signifi-cantly different from normal control (NC), *p* < 0.01; * and ** significantly different from pathogenic *Escherichia coli*, *p* < 0.05 and < 0.01, respectively.

To explore the effect of GTE on proliferation, the expression of PCNA and CD44 was assessed (Figure 7A). Decreased proliferation-related protein expression was observed in the pathogenic *E. coli* alone group. In contrast, the GTE-treated group showed an enhanced protein expression with an increased amount of CD44.

Moreover, as shown in the IHC assessment, the decreased expression of Ki-67 after pathogenic *E. coli* challenge was improved in the GTE-treated group, which showed a positive stained area in the simple columnar epithelium (Figure 7B).

## 3. Discussion

There is a growing interest in the bioactivities of phytochemicals, and polyphenols are one of the most studied and widely distributed groups of bioactive molecules. In this study, we described the biological effects of standardized GTE administration against pathogenic *E. coli*-induced colitis in mice.

The catechins from green tea are incorporated into the bacterial cell membrane, disrupting bacterial membrane barrier activity, and EGCG can effectively form hydrogen bonds with the lipids in bacterial cell membranes [27,28]. The present GTE successfully inhibited the growth of pathogenic *E. coli* in a concentration- and time-dependent manner. Moreover, incubation with 60 mg/mL GTE resulted in a more than 3-fold reduction, which indicates the strong bactericidal effect of GTE. The effective GTE concentration obtained in this study seems to be relatively higher than that obtained from the antibacterial study with chemical compounds. However, this might be due to the characteristics of natural compound crude extract, which contains relatively low active ingredient levels in comparison to the pure chemical substance, and the nature of isolated microbes, which have a high incidence of drug resistance. Moreover, although the exact comparison of tea extract potency is difficult due to the difference in extract preparation and the method employed, many studies reported MIC values of over 100 mg/mL with GTE against isolated *E. coli* [29,30]. In this study the MIC value of GTE against pathogenic *E. coli* was 40 mg/mL.

The intestinal epithelium forms a selective barrier that plays an important role in regulating mucosal homeostasis against various stimuli [31]. Following injury, the intestinal epithelium and immune cells activate the repair process to maintain membrane homeostasis, which is highly regulated by protein and lipid mediators such as Anex1 [32]. Anex1 is a glucocorticoid-induced protein that inhibits phospholipase A2 activity, thereby suppressing eicosanoid synthesis and inflammatory processes [33]. Several studies focused on the anti-inflammatory effect of Anex1 in the treatment of various inflammatory bowel diseases and revealed its therapeutic potency through suppressed inflammatory signals [34,35]. Previously, Babbin et al. [36] reported that aggravated intestinal damage in an Anex1 knockout mouse model was followed by suppressed ALX/FPRL-1 under dextran sulfate sodium-induced colitis conditions, which suggests the importance of Anex1 in modulating intestinal inflammation and mucosal injury. Furthermore, Anex1 is recognized as a pro-resolving mediator that facilitates the resolution of inflammation and mucosal wound repair [37,38]. The increased Anex1 expression, present in the GTE-treated group, might suppress the inflammatory process and improve *E. coli*-induced intestinal injury. Consistent with previous studies, the Anex1 expression observed in this study markedly increased in the GTE-treated group; therefore, increased Anex1 expression was accompanied by suppressed inflammation-related signal expression levels, including those of TNF-α, COX-2, and iNOS, which participated in the initiation and prolongation of inflammatory processes [39].

The virotype of the present *E. coli* was F18:LT:STa:Stx2e, which contains various types of toxins. In this study, we expected bloody stools after an *E. coli* challenge; however, the infected group showed significant weight loss with diarrhea rather than hemorrhagic diarrhea. Given previous studies, which used different species of isolated pathogenic *E. coli* in mice, the present absence of hemorrhagic diarrhea may be attributed to the inoculation condition or duration rather than the origin of the pathogenic *E. coli* [40,41].

A previous study by Chang et al. [20] reported that catechin inhibits enterotoxin binding to cholesterol, which is an essential initial step in causing intestinal injury through altering the secondary structure of LT subunit. The effect of catechin on LT might, at least in part, participate in the amelioration of pathogenic *E. coli*-induced pathological changes in the intestine. The main sites of colonization differ between ETEC and enterohemorrhagic *E. coli*, which are the upper jejunum and colon, respectively [42,43]. Shorter villi and deeper crypts in the jejunum are a typical phenotype of ETEC, which indicates aggravated intestinal absorption and secretion [44]. In this study, GTE treatment ameliorated pathogenic *E. coli*-derived histopathological alterations, with improved villi height and crypt depth. Moreover, increased proliferation-related protein expressions, such as those in PCNA and CD44, were enhanced in the GTE groups, which were further supported by restored Ki-67 expression in the damaged area, as evidenced by the IHC study. Intestinal epithelium homeostasis is maintained by the continuous and rapid replacement of differentiated cells by replication or cell transition [45]. The upregulated proliferation-related proteins might contribute to the improved intestinal structures in the GTE-treated group.

The present study demonstrated the prophylactic effect of GTE against pathogenic *E. coli*-induced intestinal damage. However, our study design has some limitations. The present GTE was not administered during the *E. coli* challenge period as it was judged as more suitable for evaluating the effect of GTE in preventing intestinal damage caused by pathogenic *E. coli*. However, this might hinder the clear demonstration of comprehensive antimicrobial effects of GTE and result in a poor bacterial reduction in the present GTE-treated mice. Therefore, further study with extended GTE administration periods after *E. coli* infection are required to fully determine antimicrobial activity of GTE in the body system.

## 4. Materials and Methods

### 4.1. GTE Preparation

Dried green tea was purchased from Dajayeon (Sacheon, Korea). Green tea leaves were ground to a powder, and the powder was extracted with 70% ethanol. For ethanol extraction, the powder (1.2 kg) was soaked in ethanol (12 L) and incubated for 24 h at room temperature. The extraction was repeated three times, and the upper layer was filtered and concentrated using a rotary evaporator at 40 °C (Heidolph, Schwabach, Germany). After evaporation, the extract was freeze-dried with HyperCOOL 3110 (Hanil Scientific Inc., Seoul, Korea), and the extraction yield was 32.26%.

### 4.2. HPLC Analysis

Standard compounds, catechin, epigallocatechin, and EGCG (>95% purity) were purchased from Sigma-Aldrich (St. Louis, MO, USA). The quantitative analysis of components in the GTE was performed using an HPLC coupled with an ultraviolet detector (1260 Infinity II LC System, Agilent, Palo Alto, CA, USA), and the peaks were confirmed in comparison with the reference standard peaks, in terms of the retention time and consistency. Chromatographic separation was achieved using a Hypersil BDS-C18 column (5 μm, 150 × 4.6 mm) (Thermo Fisher Scientific, Leicester, UK). The mobile phase consisted of 0.1% trifluoroacetic acid (TFA) in distilled water (A) and 0.1% TFA acetonitrile (B), and the gradient conditions were as follows: 0–1 min (100–0% A), 1–15 min (100–72% A), 15–17 min (72–72% A), and 17–30 min (72–100% A) at a flow rate of 0.45 mL/min. The injection volume was 5 μL, and the wavelength was set to 280 nm.

### 4.3. Antibacterial Activity Assessment

Pathogenic *E. coli* (KVCC-BA0001423), which had the F18:LT:STa:Stx2e virotype, was obtained from the National Veterinary Research and Quarantine Service (Gimcheon, Korea) and cultured in nutrient broth. The minimum inhibitory concentration was determined by using standard microdilution technique with some modifications (Clinical and Laboratory Standards Institute, 2012). Pathogenic *E. coli* (5 × 10^5^ CFU/mL) was incubated with serial concentration of GTE at 38 °C for 24 h. The MIC was set at lowest concentration where the visible growth of bacteria was inhibited. The bacterial colony reduction after GTE treatment was confirmed by the broth microdilution method in 96-well cell culture plates. In brief, 1 × 10^8^ CFU/mL pathogenic *E. coli* were incubated with a serial concentration of GTE containing nutrient broth at 38 °C. After different incubation times (19, 24, and 31 h), bacterial counts were determined by agar spreading. Countable serial 10-fold dilutions were prepared and 100 μL of each dilution was spread on m-TEC ChromoSelect selective agar (Sigma Aldrich) plates, and CFU were counted after overnight incubation at 38 °C. The antibacterial effect of GTE was assessed by a CFU log reduction in the GTE-treated sample, in comparison with the CFU value from the untreated positive control sample.

### 4.4. Animal Study

Five-week-old male Balb/c mice (18–20 g, *n* = 24, Orient Bio Inc., Seungnam, Korea) were housed under standard conditions (temperature, 22 ± 3 °C; humidity, 23 ± 5%; 12 h light/dark cycles) with standard laboratory chow and water provided ad libitum. All mice were acclimated for 1 week before the experiments. Animal care and experimental procedures were performed according to the guidelines of the Animal Care and Use Committee of Chungnam National University (202003A-CNU-042).

Mice were randomly divided into four groups: control, pathogenic *E. coli* alone, low-dose GTE, and high-dose GTE groups (6 mice/group). During the first 10 days, 500 and 1000 mg/kg GTE was administered by oral gavage to the low-dose and high-dose GTE groups, respectively, and phosphate-buffered saline was administered as a vehicle control. On day 9, all mice received streptomycin (20 mg/kg) to eradicate normal gut bacterial flora. After 24 h of fasting, the freshly prepared pathogenic *E. coli* (KVCC-BA0001423, 100 μL, 1 × 10^9^ CFU/mL) was administered orally to the pathogenic *E. coli* alone, low-dose GTE, and high-dose GTE groups for three consecutive days. All mice were sacrificed on the third day, after the last treatment, with carbon dioxide, and the jejunum tissue was harvested for Western blot and histopathological analysis. The colon tissue was collected in sterile saline, and the bacterial count was determined by spreading each dilution on ChromoSelect selective agar.

### 4.5. Histopathological Analyses

Formalin-fixed jejunum tissues were dehydrated and embedded in paraffin. Paraffin sections (4 μm) were processed and stained with hematoxylin and eosin. The morphological characteristics were observed under a light microscope (Leica, Wetzlar, Germany) at a magnification of 200×. For histopathologic scoring, all slides were scanned using a digital slide scanner (MoticEasyScan Pro, Motic, Xiamen, China). The height and depth of villi and crypts were measured in 10 randomly selected microscopic fields using the Motic Digital Slide Assistant software (version 1.0.7.61, Motic) and the ratio between the lengths of the two structures was calculated [46].

### 4.6. Western Blot Analysis

The frozen intestine tissue was homogenized (1:9, *w*/*v*) with Lysis buffer (Sigma-Aldrich) containing a protease inhibitor cocktail (Sigma-Aldrich) and a phosphatase inhibitor cocktail (Sigma-Aldrich), and centrifuged at 12,000× *g* at 4 °C for 20 min to isolate the cellular proteins in the supernatant. To investigate protein expression related to apoptotic changes, we performed Western blotting according to a previous study [47]. After blocking with bovine serum albumin blocking buffer, membranes were incubated with various primary antibodies, such as anti-Annexin A1 (Anex1, 1:1000, Abcam, Cambridge, MA, USA), anti-formylpeptide receptor-like 1 (ALX/FPRL-1, 1:1000, Abcam), anti-inducible nitric oxide synthase (iNOS, 1:1000, Abcam), anti-cyclooxygenase 2 (COX-2, 1:1000, Abcam), anti-TNF-α (1:1000, Abcam), anti-proliferating cell nuclear antigen (PCNA; 1:1000, Abcam), anti-CD44 (1:1000, Abcam), and β-actin (1:1000, Abcam). After washing and adjusting the secondary antibody according to the manufacturer’s protocol, quantitative analysis of each protein band was performed using ChemiDoc (Bio-Rad Laboratories, Hercules, CA, USA).

### 4.7. IHC Analysis

After deparaffinization and dehydration, the sections were treated with 0.5% Triton X-100 solution for 30 min at room temperature. After serial washing, endogenous peroxidase quenching, and blocking, the sections were incubated overnight with the following primary antibodies: anti-Anex1 (1:200, Abcam), ALX/FPRL-1 (1:200, Abcam), anti-COX-2 (1:200, Abcam), and anti-Ki-67 (1:200, Abcam). The VECTASTAIN Elite ABC kit (Vector Laboratories, Burlingame, CA, USA) and 3,3′-diaminobenzidine (DAB) were used for color development. After counter-hematoxylin staining, all sections were randomly evaluated under a light microscope (Nikon Eclipse 80; Nikon Corporation, Tokyo, Japan).

### 4.8. Statistical Analyses

Data are shown as mean ± standard deviation (SD). Means of more than two groups were analyzed via one-way analysis of variance followed by Dunnett’s multiple comparisons test. Statistical analyses comparing the treatment groups to the vehicle control group were performed using an unpaired *t*-test (GraphPad Software, Inc., La Jolla, CA, USA). Statistical differences were considered significant at *p* < 0.05.

## 5. Conclusions

In the present study, GTE was found to have an antibacterial activity against the wild-type pathogenic *E. coli* and ameliorated pathogenic *E. coli*-induced intestinal damage by modulating inflammation and epithelial cell proliferation. Further mechanistic studies are required to reveal the exact correlation between green tea catechins and Anex1 in pathogenic *E. coli* infections.

## Figures and Tables

**Table 1:** Antimicrobial effect of catechin compounds from green tea.

Compound	Pathogens	Signals (Marker)	References
Epigallocatechin	*Escherichia coli*; *Escherichia coli* O157:H7; *Escherichia coli*; *Pseudomonas aeruginosa*; *Escherichia coli*	ATP and potassium pool; Verotoxin; Cellular defense protein; Antibacterial Susceptibility; Antibacterial Susceptibility	[14,15,16,17,18]
Epicatechin	*Escherichia coli*; *Escherichia coli* O157:H7; *Escherichia coli*; *Pseudomonas aeruginosa*; *Escherichia coli*; *Gram-positive bacteria*; *Aggregatibacter actinomycetemcomitans*	ATP and potassium pool; Verotoxin; Cellular defense protein; Antibacterial Susceptibility; Antibacterial Susceptibility; Bacterial membrane; Leukotoxin activity	[14,15,16,17,18,19,20]
Gallocatechin gallate	*Escherichia coli* O157:H7; *Escherichia coli*; *Pseudomonas aeruginosa*	Verotoxin; Cellular defense protein; Antibacterial Susceptibility	[15,16,17]
Catechin	*Escherichia coli* O157:H7	Verotoxin	[15]
Epigallocatechin gallate	*Escherichia coli*; *Pseudomonas aeruginosa*; *Escherichia coli*; *Gram-positive bacteria*; *Escherichia coli*; *Bacillus subtilis*; *E. coli* MG1655; *Pseudomonas fluorescens*; *Staphylococcus aureus*	Cellular defense protein; Antibacterial Susceptibility; Antibacterial Susceptibility; Bacterial membrane; H_2_O_2_; Cell surface proteins; Hfq protein; Extra-cytoplasmic function sigma factor, Histidine kinase; Membrane transport	[16,17,18,19,21,22,23,24,25]
Epicatechin gallate	*Escherichia coli* O157:H7; *Escherichia coli*; *Pseudomonas aeruginosa*; *Escherichia coli*; *Escherichia coli*; *Staphylococcus aureus*	Vero toxin; Cellular defense protein; Antibacterial Susceptibility; H_2_O_2_; β-lactam resistance	[15,16,17,18,21,26]

## Data Availability

The datasets generated during and/or analyzed during the current study can be find in the main text.
